# Sleep-Disordered Breathing and Interactions with Opioids: A Narrative Review

**DOI:** 10.3390/jcm14134758

**Published:** 2025-07-04

**Authors:** Peyton J. Murin, Jora Wang, Yuri Chaves Martins

**Affiliations:** 1 Department of Neurology, Saint Louis University School of Medicine, St. Louis, MO 63104, USA; 2 College of Medicine, Saint Louis University School of Medicine, St. Louis, MO 63104, USA; jora.wang@health.slu.edu; 3 Department of Anesthesiology, Saint Louis University School of Medicine, St. Louis, MO 63110, USA

**Keywords:** sleep-disordered breathing, obstructive sleep apnea, central sleep apnea, opioid use, chronic pain

## Abstract

Opioid use in patients with sleep disordered breathing (SDB) presents therapeutic challenges within chronic pain and sleep medicine. Opioids impair respiratory drive through μ-opioid receptor activation in brainstem respiratory centers, exacerbating both obstructive and central apneas. Chronic opioid use is also linked to a high prevalence of central sleep apnea and increased nocturnal hypoventilation. Simultaneously, SDB contributes to heightened pain sensitivity via intermittent hypoxia, systemic inflammation, and alterations in neural plasticity. These mechanisms may influence opioid efficacy and dosing requirements. This review summarizes current evidence on how SDB and opioid use interact, emphasizing chronic opioid use in the setting of chronic pain management. We discuss the underlying mechanisms, clinical impacts, and potential avenues for enhanced diagnosis and therapy in this population. We conclude that the intersection of SDB and opioid use presents a complex clinical challenge that demands a multidisciplinary approach. Enhanced screening, personalized pharmacologic strategies, and integration of advanced diagnostics are essential for mitigating risks and optimizing care. Future research should focus on mechanistic studies and interventional trials to guide evidence-based management of this high-risk population.

## 1. Introduction

Sleep-disordered breathing (SDB)—including obstructive and central sleep apnea—lies at a crucial crossroads in sleep medicine and pain management, with major implications for patient safety and treatment success. While the impact of illicit opioid use is well-established, there is increasing evidence to suggest that chronic use, even under medical guidance, can lead to adverse health outcomes [1]. Uncontrolled pain results in decreased sleep quality. However, recent work has shown this effect may be bidirectional, with poor sleep quality leading to worsened pain [2,3]. This is supported by the improvement in pain with treatment of SDB [4]. At the same time, the respiratory depressant effect of opioids has a deleterious effect on the pathophysiology of SDB [5]. This is compounded by suppression of central and peripheral chemoreceptor sensitivity [6]. As a result, this creates a vicious cycle where SDB worsens pain and treatment of pain with opioids worsens SDB. At a population level, this results in heightened perioperative risks, altered analgesic efficacy, and increased mortality rates [7].

The clinical urgency surrounding this topic is underscored by the concurrent rise in the prevalence of opioid use [8] and undiagnosed SDB [9]. Post operative opioid prescriptions have continued to rise, with one study noting an increase of 80–145% in mean milligram morphine equivalents for lumbar laminectomy/laminotomy, total knee arthroplasty, and total hip arthroplasty [10]. While the intention is for these to be short courses of acute opioid use, there is an increased risk for persistent opioid use, with >60% of patients receiving 90 days of opioids transitioning to chronic use [11]. Furthermore, opioid prescribing seems to be increasing fastest amongst elderly patients [12], a cohort at increased risk of SDB [13]. Simultaneously, the prevalence of untreated SDB continues to represent a major health burden [14]. In the United States, it is estimated that 20% of the adult population suffers from SDB, with a prevalence of ~33% in patients 50 to 70 years old [15]. While there is significant literature showing the association of untreated SDB with cardiovascular disease [16], chronic obstructive pulmonary disease [17], and stroke [18], the impact on chronic pain and chronic opioid use is less well understood.

Addressing the complex interplay between SDB and opioid use, especially in patients with chronic pain, requires a multidisciplinary strategy integrating anesthesiology, pulmonology, pain management, and sleep medicine. This review explores the prevalence, underlying mechanisms, and clinical consequences of SDB in opioid users, with an emphasis on chronic opioid use in chronic pain management, providing a broad analysis to support future research and practice.

## 2. Materials and Methods

This narrative review investigates the two-way relationship between SDB and opioid use, focusing on biological mechanisms, clinical outcomes, and relevance for chronic pain management. To achieve this, a comprehensive literature search was performed using the PubMed, Scopus, and Web of Science databases for peer-reviewed articles published in English up to June 2025. Keywords and Medical Subject Headings (MeSH) included combinations of the following terms: “sleep-disordered breathing”, “obstructive sleep apnea”, “central sleep apnea”, “opioids”, “opioid-induced respiratory depression”, “pain sensitivity”, and “chronic pain”. Actively recruiting clinical trials were also searched at www.clinicaltrials.gov.

Studies were included based on their relevance to understanding the physiology, epidemiology, drug interactions, and treatments related to SDB in opioid users. Additional inclusion criteria were systematic reviews, randomized controlled trials, cohort studies, and foundational experimental research in humans and animals. Bibliographies of key articles were manually screened to identify further pertinent studies not retrieved in the primary search.

Data were synthesized qualitatively with a focus on identifying themes related to (1) SDB prevalence and demographics; (2) how opioids affect breathing and sleep; (3) how SDB influences opioid effectiveness and pain perception; and (4) new diagnostic and treatment strategies. Figures were included to conceptualize mechanistic pathways and clinical consequences where appropriate. Emphasis was placed on integrating multidisciplinary perspectives from anesthesiology, sleep medicine, pulmonology, and pain management.

This review did not involve primary data collection or statistical analysis and thus was exempt from institutional review board approval.

## 3. Results

### 3.1. How Common Is Sleep-Disordered Breathing?

The prevalence of SDB is both significant and heterogeneous across populations. Polysomnography remains the definitive diagnostic modality, particularly for complex presentations involving mixed apneas or overlapping comorbidities [19]. Obstructive sleep apnea (OSA), the most common form of SDB, affects an estimated 9–38% of the general adult population [20]. This prevalence increases in high-risk groups, such as individuals with obesity (over 50%) and older adults (up to 80%) [21]. In pediatric populations, OSA prevalence ranges between 1–5%, often manifesting with behavioral and developmental sequelae if untreated [22]. Central sleep apnea (CSA), though less prevalent, is particularly associated with chronic opioid use, heart failure, and cerebrovascular conditions [23,24].

Importantly, patients with OSA are more likely to receive opioids. Even after adjusting for comorbidities and demographics, studies show that patients with OSA are more likely to be prescribed opioids both chronically [relative risk reduction (RRR) = 2.15 (95% CI = 2.09–2.22)] and acutely [RRR = 2.02 (95% CI = 1.98, 2.06)] [25].

**Figure 1:**
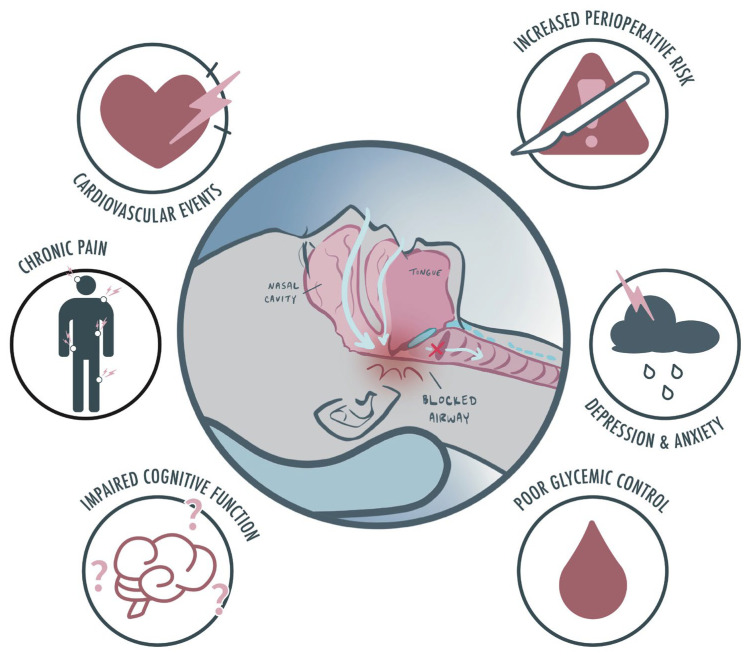
Sleep apnea and comorbidities: There is an established association between sleep disordered breathing and cardiovascular events, impaired cognitive function, poor glycemic control in diabetes, mood disorders, chronic low back pain, and increased perioperative risk when undergoing procedures. This highlights the importance of identifying and treating sleep-disordered breathing from a public health and patient care perspective.

The hidden burden of undiagnosed SDB in surgical populations is substantial. In a seminal study, Finkel et al. [26] revealed that 82% of high-risk surgical patients previously undiagnosed with OSA were identified via screening tools such as the STOP-Bang questionnaire, which boasts a sensitivity of 94% and specificity of 79%. The global ramifications of untreated SDB include a two- to three-fold increase in cardiovascular events [27], poor glycemic control in diabetes [28], impaired cognitive function [29], mood disorders [30], chronic low back pain [31], and heightened perioperative risks [32], all of which contribute to considerable economic and healthcare system burdens (Figure 1).

Geographic and demographic trends also shape SDB prevalence. For instance, the condition is more prevalent in industrialized nations due to higher rates of obesity and sedentary lifestyles [14].

### 3.2. What Are the Effects of Opioids on Sleep-Disordered Breathing?

**Figure 2:**
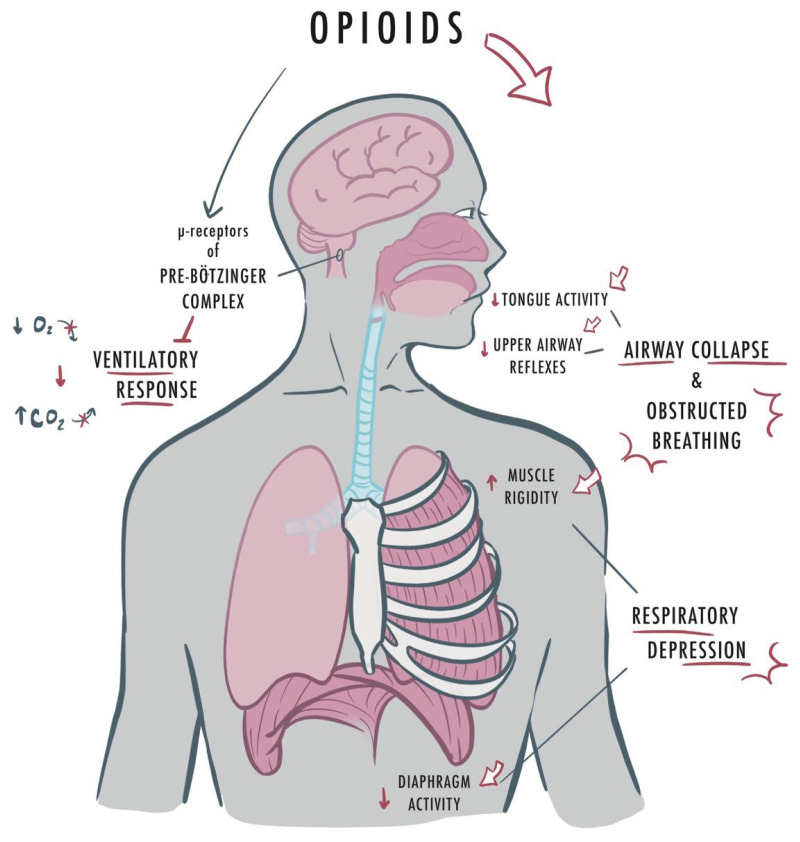
Opioid effects on the airway: Opioids have several deleterious effects on the respiratory system. Opioids act on the pre-Bötzinger complex to decrease ventilatory response, decrease tongue activity, upper airway reflexes, and diaphragm activity, while simultaneously increasing muscle rigidity. This all acts to worsen the effects of pre-existing sleep-disordered breathing and increase morbidity, highlighting the importance of diagnosis and management.

The exact mechanisms of the harmful interactions between opioid use and SDB pathogenesis remain poorly understood [33]. Opioid-induced respiratory depression (OIRD) is exacerbated by coexisting SDB [34]. Opioids exert profound effects on respiratory physiology by acting on μ-opioid receptors within the brainstem, particularly the pre-Bötzinger complex [35], a key respiratory rhythm generator. This interaction diminishes ventilatory responses to hypercapnia and hypoxemia, thereby exacerbating both OSA and CSA. Acute administration of opioids, such as remifentanil, can precipitate central apneas and irregular respiratory patterns. For example, Bernards et al. demonstrated that short-term opioid infusions in individuals with moderate OSA led to significant respiratory depression and increased apnea-hypopnea index (AHI) [36]. More recently, it was shown that opioid use is associated with higher loop gain (unstable ventilatory control) and increases respiratory rate variability in patients with SDB [33]. Acute opioid use also suppresses upper airway reflexes, further contributing to airway collapsibility and obstruction [37] (Figure 2).

Chronic opioid therapy heightens these effects, leading to a high prevalence of CSA, reported in up to 55% of long-term opioid users [38]. Compared to controls, patients with chronic opioid use are more likely to have central apneas (2.8 versus 1.7 events) and low oxygen saturation (5% versus 3% with SpO_2_ < 88%) [33]. Dose-dependent effects are evident, with patients on daily morphine milligram equivalents (MME) above 200 exhibiting severe CSA and ataxic breathing patterns [39]. Methadone, a long-acting opioid, is associated with sustained hypoventilation due to its prolonged clearance, whereas buprenorphine, a partial agonist, appears to offer a ceiling effect on respiratory depression, potentially making it a safer alternative [40]. Fentanyl and remifentanil, commonly used in perioperative settings, are potent suppressors of respiratory drive, with rapid onset and short duration of action, making them high-risk agents for the development of hypoxia. Hydromorphone and oxycodone, frequently prescribed for chronic pain, display longer-lasting effects, and thereby increase the likelihood of nocturnal hypoventilation and sleep disruption. Comparative studies of these agents in populations with pre-existing SDB are warranted to refine clinical guidelines. In addition, genetic factors further modulate the impact of opioids on respiratory function. Polymorphisms in the OPRM1 gene, encoding the μ-opioid receptor, have been linked to variability in respiratory depression and opioid sensitivity [41]. These findings underscore the necessity for precision medicine approaches in managing opioid-induced SDB. Additionally, advances in pharmacogenomics may pave the way for individualized opioid prescribing that mitigates respiratory risks while optimizing analgesia.

### 3.3. What Are the Effects of Sleep-Disordered Breathing on Opioid and Pain Sensitivity?

**Figure 3:**
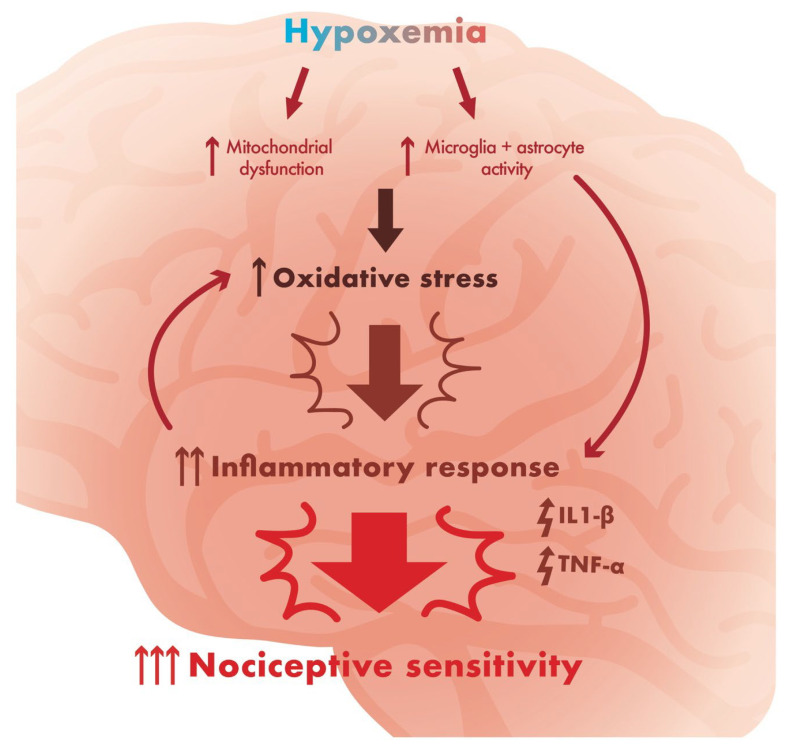
Pathogenesis of sleep, chronic inflammation, and pain: Sleep-disordered breathing results in a state of chronic hypoxemia. This results in oxidative stress, leading to increased levels of inflammatory mediators such as IL-6 and TNF-α, resulting in a chronic inflammatory response and increased nociceptive sensitivity. This has a deleterious result on pain control, possibly resulting in higher doses of opioids needed to achieve a therapeutic response and a higher risk of side effects.

The bidirectional relationship between SDB and pain sensitivity involves intricate pathophysiological processes, including hypoxemia, systemic inflammation, and disrupted sleep architecture [3]. The relationship between hypoxia and inflammation is well established, with patients displaying increased levels of inflammatory markers in lower oxygen states (such as mountain sickness) [42]. In low oxygen states, the hypoxia-inducible factor (HIF) α and HIF-β subunits will bind to a hypoxia response promoter element (HRE). This binding upregulates transcription of genes for inflammatory markers such as nuclear factor κB (NF-κB) [42]. This may result in a state of chronic low-grade inflammation [43], potentially contributing to persistent, refractory chronic pain (Figure 3). Chronic intermittent hypoxemia, a hallmark of SDB, activates inflammatory pathways. This is supported by previous literature identifying elevated cytokines such as interleukin-1β (IL-1β) and tumor necrosis factor-α (TNF-α) in patients with SDB [43,44]. In addition, higher levels of IL-6 and TNF-α correlate with neuronal damage and patient-reported levels of bodily pain in the same population of patients [43,45]. These findings are corroborated by studies showing that intermittent hypoxia causes neuronal inflammation and neural plasticity changes in the brain and spinal cord of mice [46].

Sleep fragmentation also impacts pain sensitivity. Previous literature has noted that sleep fragmentation results in reduced pain tolerance and pain thresholds [47]. Furthermore, there is some evidence to suggest an association between pain intensity and sleep quality [48]. There was an increased risk of chronic low back pain and back pain-associated disability in patients with chronic poor sleep quality when compared with patients with healthy sleep [31]. Importantly, this effect may extend beyond the initial insult. Prolonged exposure to elevated levels of pro-inflammatory factors (IL-6, TNF-α, and chemokines) can result in alterations within synaptic connections and neuronal signaling pathways [43,45], with microglia proposed to be a key mediator of this plasticity [49]. This has been linked to changes in pain processing centers and central sensitization [50], resulting in chronic augmented pain sensitivity [51,52,53]. This effect is not just limited to the brain itself. Recent work has identified cellular senescence within the dorsal root ganglion occurring following exposure to IL-6 [54], suggesting that chronic low-grade inflammation may impact the dorsal root ganglion as well. These novel mechanistic links underscore that chronic hypoxemia in opioid-treated SDB patients could further amplify inflammation and oxidative stress, contributing to hyperalgesia and end-organ damage.

### 3.4. What Are the Effects of Genetics on Opioid Sensitivity and Sleep-Disordered Breathing?

The interplay of genetics and opioid response is particularly critical in patients with SDB. Polymorphisms in genes like COL11A1 (connective tissue and pain sensitivity), COMT (pain modulation enzyme), CYP2D6 (drug metabolism), OPRM1 (μ-receptor), and other genes further influence individual variability in pain thresholds and opioid responsiveness, highlighting the importance of genetic predispositions [55,56,57].

The OPRM1 A118G single-nucleotide polymorphism (rs1799971) is common but varies by ethnicity. The minor 118G allele has a frequency of roughly 11–17% in Caucasian populations and 27–48% in Asian populations, but is much rarer in African Americans (~2%) and Sub-Saharan Africans (<1%) [56]. Approximately 25–40% of Asians carry the G allele, compared to ~10–15% of individuals of European descent [56]. This makes the variant an important consideration across diverse patient groups. For SDB patients, the A118G polymorphism raises special concerns. G-allele carriers may experience less opioid analgesia, leading to escalated dosing that can precipitate respiratory depression. Notably, experimental anesthesia studies show that while 118G homozygotes get significantly less analgesia from opioids like morphine-6-glucuronide, they are not protected from respiratory depressive effects [57]. In other words, the 118G variant differentially affects analgesia vs. breathing—analgesic potency is reduced, but the toxic effects (like respiratory depression) remain just as potent [57]. Thus, an OSA patient with the 118G allele might require higher opioid doses to control pain, yet still suffer normal (or even heightened) respiratory depression at those doses. Indeed, one study concluded that the OPRM1 118G SNP “does not protect against the toxic [respiratory] effects” of opioids despite blunting analgesia [58]. Clinically, this means such patients are at high risk for opioid-induced ventilatory impairment and should be closely monitored. They may benefit from multimodal analgesia or alternative agents to avoid simply “dose-stacking” opioids. There is also evidence that chronic intermittent hypoxia in OSA can upregulate opioid receptors (via HIF-1α) and increase opioid sensitivity [57]. This hypoxia-induced increase in μ-receptor expression, coupled with an OPRM1 variant, could further tilt OSA patients toward both enhanced analgesic response and greater respiratory suppression. Therefore, in SDB patients carrying OPRM1 118G, clinicians should titrate doses cautiously, augment monitoring (e.g., capnography), and consider opioid-sparing strategies [57].

The COMT Val158Met SNP (rs4680) is another common variant with ethnic variability. In many European populations, the Met allele frequency is around ~50% (meaning roughly half of individuals carry at least one Met^158 allele) [59]. By contrast, East Asian and African groups have a higher prevalence of the Val allele (the high-activity form) and correspondingly fewer Met allele carriers [60]. For example, the Met^158 allele frequency in East Asians can be as low as ~20% or less [59], and the homozygous Met/Met genotype is relatively uncommon in those populations. In Caucasians, about 20–25% of people are Met/Met, 50% Val/Met, and 25–30% Val/Val, whereas in Asians the Val/Val genotype predominates [60]. This variation implies that the impact of COMT on pain and opioid response may differ across ethnic groups. In SDB patients, the COMT Val158Met polymorphism may indirectly heighten opioid risks by affecting pain and dose requirements. A Met/Met patient tends to have more pain (or less endogenous pain inhibition) and thus may demand larger opioid doses to achieve comfort [61,62]. In the context of SDB, higher opioid dosing is perilous—it increases the likelihood of severe oxygen desaturation or even apnea-induced arrest. Thus, an OSA patient with the Met/Met genotype could be considered a “high pain, high opioid need” phenotype, meriting aggressive use of non-opioid modalities (NSAIDs, nerve blocks, etc.) to limit opioid consumption. Additionally, some evidence suggests COMT Met allele carriers might experience somewhat diminished opioid-induced euphoria and analgesia, potentially prompting an increase in dose if a provider is unaware of their genotype [62]. Conversely, Val/Val individuals (low pain sensitivity) might achieve pain relief with lower opioid doses, which could be safer in SDB. Although the COMT genotype alone is not a determinant of respiratory depression, its effect on required opioid dose is clinically relevant, as any polymorphism that drives up opioid dosing will magnify respiratory risk in SDB patients [63]. In practice, patients with known COMT Met/Met (if tested) should be flagged for opioid-sparing pain management. Moreover, untreated OSA itself can alter pain processing—chronic intermittent hypoxia and inflammation in OSA may activate pronociceptive pathways but also induce some antinociceptive adaptation (e.g., increased opioid receptor expression) [58]. The net result in OSA is often an increased sensitivity to opioids’ effects [58]. Thus, when a high-pain genotype (COMT Met/Met) coexists with OSA, the patient should be treated as high-risk: closely monitor sedation level and respiratory status and consider lower starting doses than typical.

COL11A1, encoding the α1 chain of type XI collagen, is not a traditional “opioid gene,” but certain polymorphisms in COL11A1 have emerged as significant in pain and possibly in SDB contexts. One functional SNP in COL11A1 (c.4603C>T, rs1676486) was identified in Japanese populations with a minor allele frequency sufficient to confer a notable association with lumbar disc disease [64]. This T variant (minor allele) is common enough (in East Asia) to explain a fraction of disc herniation cases [61], though exact population frequencies vary. Generally, COL11A1 variants linked to musculoskeletal pain tend to be moderately prevalent (e.g., 10–30% allele frequency), so their impact can be seen at the population level. There is less data on COL11A1 allele frequency in Western populations; however, meta-analyses confirm that rs1676486 is significantly associated with intervertebral disc degeneration across studies [65]. For patients with SDB, COL11A1 polymorphisms are relevant mainly insofar as they predispose to chronic pain conditions that necessitate opioid therapy. An OSA patient who carries the COL11A1 risk allele may be more likely to suffer severe back pain or osteoarthritis, thus ending up on long-term opioids. This is a dangerous intersection as OSA + chronic opioid use greatly heightens the risk of fatal respiratory depression during sleep [66]. Clinicians should recognize that a patient’s genetics might make them inherently more pain-prone. For example, if a COL11A1 variant contributes to unremitting back pain, simply prescribing higher opioid doses is a poor solution, especially in OSA. Instead, such patients could benefit from targeted non-opioid treatments for their underlying condition (e.g., early disc intervention, physiotherapy, anti-inflammatory biologics in arthritis). Additionally, because type XI collagen is present in soft tissue airway, one could hypothesize that COL11A1 variants might influence pharyngeal collapsibility in OSA (collagen defects could mean floppier airways). While direct evidence is limited, any genetic collagen weakness could exacerbate OSA severity and thus further reduce the margin of safety for opioid dosing. In practical terms, if an SDB patient is known (or suspected) to have a COL11A1-related pain vulnerability (e.g., family history of disc disease), clinicians should anticipate higher analgesic needs and plan accordingly. For example, by using spinal blocks for surgical pain, avoiding muscle relaxants that could worsen airway collapsibility, and vigilantly monitoring breathing when opioids are given. Therefore, the overlap of connective tissue genotype, chronic pain, and OSA creates a scenario where personalized pain management is essential.

The cytochrome P450 2D6 gene (CYP2D6) is highly polymorphic, and variant alleles produce a spectrum of metabolic phenotypes: poor, intermediate, extensive (normal), or ultrarapid metabolizers (UMs). Approximately 5–10% of Caucasians have two nonfunctional alleles (poor metabolizers), leading to little or no CYP2D6 activity [67]. In East Asian populations, poor metabolizers are slightly less common (~1–2%), whereas intermediate metabolizers are more frequent due to the prevalence of reduced-function alleles (like *10). UMs carry gene duplications that amplify CYP2D6 activity. UMs are relatively rare in Northern Europe (~1–2%) but much more common in certain ethnic groups—for example, up to ~10% of Mediterranean and 16–28% of North African and Middle Eastern populations have UM genotypes [68]. Notably, one meta-analysis predicted that 1% to as high as 21% of individuals worldwide could be UM depending on the ethnic cohort [69]. In short, the likelihood of encountering an ultra- or poor metabolizer is not negligible, and identifying these extremes is crucial when prescribing opioids that are CYP2D6 substrates. CYP2D6 polymorphisms have direct and dramatic implications for SDB patients on opioids. The textbook scenario is the child with OSA who, after adenotonsillectomy, is given codeine for pain and suffers apnea or death because they are a UM. In fact, multiple case reports and an FDA safety review documented deaths in children with OSA who took codeine post-tonsillectomy and were later found to have CYP2D6 UM genotypes [70,71]. These children metabolized codeine to morphine extremely rapidly, resulting in toxic morphine blood levels and fatal respiratory depression during sleep [72]. As a result, regulatory agencies now contraindicate codeine (and even tramadol) in postoperative pediatric OSA patients [72]. Even in adults, an unrecognized UM can quickly accumulate high opioid concentrations. For example, an OSA adult who is a UM might experience profound hypoventilation from what is a “normal” dose for others. On the flip side, a CYP2D6 poor metabolizer with SDB might not get adequate analgesia from standard doses of drugs like codeine, potentially leading to uncontrolled pain and use of higher doses or stronger opioids, which again raises respiratory risk. Therefore, genetic testing for CYP2D6 (or choosing opioids not reliant on CYP2D6) is highly advised in SDB patients. In practice, many hospitals have moved away from codeine in children entirely, using morphine or other analgesics to avoid this genetic roulette. In adult OSA patients, if codeine or tramadol is used, one should assume variable metabolism and extensive monitoring or pre-test genotyping can improve safety. Another consideration is drug interactions—CYP2D6 inhibitors (like paroxetine) can phenocopy a poor metabolizer, causing drug buildup [65]; such interactions in an OSA patient on opioids could precipitate unexpected respiratory depression. Therefore, for SDB patients, caution should be used when prescribing opioids with CYP2D6-dependent activation (codeine, tramadol). If an OSA patient is a known UM, we suggest avoiding those opioids and using alternative drugs like morphine, hydromorphone or fentanyl, which have more predictable metabolism in this population. For known poor metabolizers, recognize that drugs like codeine will be ineffective; providing more will only add side effects without analgesia. Tailor the analgesic choice (e.g., hydromorphone or morphine that do not need CYP2D6 activation) and always err on the side of lower doses with vigilant respiratory monitoring in this population.

All the above polymorphisms reinforce a key principle for treating pain in SDB: one size does not fit all. OSA patients already have an increased baseline sensitivity to opioids’ respiratory depressant effects [58]. Genetic factors can amplify this sensitivity or increase the exposure to opioids (via higher dose needs or slower clearance). Therefore, personalizing therapy is not just ideal, it is necessary. Simple measures like avoiding codeine in known CYP2D6 UMs, or opting for regional anesthesia in an OPRM1 118G carrier with severe OSA, can prevent catastrophic outcomes.

## 4. Discussion: Unanswered Questions and Future Directions

Future investigations should adopt longitudinal designs employing advanced methodologies, such as multimodal neuroimaging (e.g., fMRI, PET) and transcriptomic profiling, to delineate the mechanistic interplay between SDB, opioid use, and pain modulation. For example, previous literature has noted an association between offset analgesia and decreased connectivity between the right medial prefrontal cortex and posterior cingulate cortex [73]. The integration of artificial intelligence algorithms with clinical data, including polysomnography and genetic markers, and imaging could enhance diagnostic accuracy for central sleep apnea (CSA) in opioid users [73]. Emerging technologies, including wearable biosensors [74] and home-based polysomnography systems [75], hold promise for enhancing real-time monitoring and personalized interventions for at-risk populations. Integration of these tools with cloud-based analytics and telemedicine platforms could revolutionize SDB management in opioid users, particularly in underserved and remote communities.

On the therapeutics side, randomized controlled trials evaluating advanced positive airway pressure (PAP) modalities, such as adaptive servo-ventilation (ASV) and bilevel PAP with backup rates, are imperative for optimizing CSA management [76]. Additionally, novel pharmacological strategies targeting opioid receptor pathways could be explored to mitigate respiratory complications while preserving analgesic efficacy. One such example includes Olicerdine, a G protein-biased ligand μ-opioid receptor agonist, potentially offering comparable analgesia with increased specificity, limiting respiratory depression [77]. Similarly, approaches have been proposed for the development of peripherally restricted opioid agonists, limiting crossing of the blood–brain barrier [78], and minimizing central effects, including respiratory depression. Alternatively, approaches have been proposed targeting other mediators in the pain pathway, such as neurokinin-1 [79] or the transient receptor potential vanilloid 1 receptor (TRPV1) [80]. TRPV1 modulation can reduce opioid-induced hyperalgesia and even opioid reward, as well as provide peripheral analgesia [80]. In addition, combining a TRPV1 antagonist with opioids showed synergistic pain relief in preclinical studies without added respiratory suppression. Another emerging frontier is the testing of interventions to mitigate opioid-related SDB. For instance, a clinical trial is examining acetazolamide (a respiratory stimulant that induces metabolic acidosis) as a treatment for opioid-induced central sleep apnea [81]). Acetazolamide has shown efficacy in high-altitude periodic breathing and heart failure-related CSA, and it is being tested to see if it can reduce apnea severity in chronic pain patients on opioids. Collaborative, interdisciplinary research consortia comprising anesthesiologists, pulmonologists, pain specialists, geneticists, and computational scientists will be critical for addressing the multifaceted challenges posed by SDB and opioid interactions. Such collaborations will facilitate translational research, bridging mechanistic insights with clinical applications to improve outcomes.

In addition, high-level evidence shows that genetic variants like OPRM1 118G and CYP2D6 poor/ultrarapid metabolizer status contribute to significant variability in opioid requirements and adverse effects [82]. A precision medicine approach—identifying these polymorphisms through genotyping (ideally preoperatively or before chronic opioid therapy)—would allow clinicians to individualize pain management strategies: selecting safer analgesics, adjusting opioid doses, and implementing strict monitoring or prophylactic respiratory support. In summary, pharmacogenetics offers a pathway to safer analgesia in SDB by accounting for each patient’s unique genetic makeup. By prioritizing such personalized care, we can relieve pain effectively while minimizing the risk of respiratory depression in this vulnerable population [58,72].

Despite growing knowledge, translating the neuroimmune and genetic findings in SDB patients with chronic pain into practical clinical guidelines remains challenging. One gap is the suboptimal implementation of OSA screening and monitoring in opioid-treated patients. Guidelines have long called for routine OSA risk screening and use of CPAP or enhanced monitoring for patients on opioids [83,84]. However, rates of postoperative OIRD and undiagnosed SDB in chronic pain patients remain concerningly high [3,85]. This indicates that clinicians face barriers in identifying high-risk patients and in ensuring compliance with therapy outside controlled trials. This problem may be solved by more holistic risk assessments and better adherence to monitoring protocols. However, another lesson is that knowledge alone is not enough without addressing uptake and feasibility in real-world settings.

## 5. Conclusions

The complex interplay between SDB and opioid therapy underscores the need for a multidisciplinary approach to patient care. Opioids exacerbate both OSA and CSA, while SDB amplifies pain sensitivity and complicates opioid management. Comprehensive screening, precision-guided opioid prescribing, and tailored therapeutic interventions, such as PAP devices, are integral to optimizing patient outcomes. Continued research and interdisciplinary collaboration will be essential in addressing the pervasive challenges posed by this critical intersection in clinical medicine.

## Data Availability

No new data were created.

## Abbreviations

The following abbreviations are used in this manuscript:

SDB	Sleep disordered breathing
CSA	Central sleep apnea
SNP	Single-nucleotide polymorphism
MeSH	Medical subject headings
OSA	Obstructive sleep apnea
OIRD	Opioid-induced respiratory depression
AHI	Apnea-hypopnea index
MME	Morphine milligram equivalents
IL-1β	Interleukin-1β
TNF-α	Tumor necrosis factor-α
Val	Valine
Met	Methionine
CYP2D6	Cytochrome P450 2D6 gene
UMs	Ultrarapid metabolizers
PAP	Positive airway pressure
